# A common law power to dissect: a medico-legal history

**DOI:** 10.1093/medlaw/fwaf006

**Published:** 2025-02-13

**Authors:** Joshua Shaw

**Affiliations:** Kent Law School, University of Kent, Canterbury, CT2 7NS, United Kingdom

**Keywords:** anatomy law, Canada, dead body disposal, law of the dead, medico-legal history, United Kingdom

## Abstract

Some jurists claimed there was a common law power to dissect the human body prior to and outside of the *Anatomy Act 1832*. That power formed part of the privileges of physicians, surgeons, and apothecaries, and, accordingly, the common law to the extent it recognized those privileges. It is best evidenced in the late-nineteenth and early-twentieth centuries—most authoritatively by the Court of Queen’s Bench in *R v Price* in 1884, the Québec Superior Court in *Phillips v Montreal General Hospital* in 1908, and the reasons of the inquiry into the conduct Dr William Ramsay Smith in 1903, but also in the comments of writers in law manuals until the mid-twentieth century. The existence of a common law power to dissect challenges narratives ordinarily told about the history of anatomy law specifically and the law of the dead generally. The power may also still exist if legislation in a jurisdiction has not displaced or substantially altered it. Through medico-legal history, the author argues that the medical lawyer can benefit from re-examining old doctrines. Heterodox elements in old doctrines suggest alternative possibilities for the law, allowing medical law’s histories to be retold.

## I. INTRODUCTION

Comments on the legal history of anatomy and dissection in common law jurisdictions often cite three statutes.^1^ First, the *Concerning Barbers and Chirurgians Act 1540*, which entitled the Company of Barbers and Surgeons of London to the bodies of four executed convicts each year for the purpose of dissection.^2^ Secondly, the *Murder Act 1751*, which granted courts the power to sentence murderers to be dissected by members of the Company of Surgeons of London after their execution.^3^ And, finally, the *Anatomy Act 1832*, which repealed the *Murder Act 1751* to the extent that it allowed dissection as punishment, empowered those in lawful possession of bodies to direct their use for anatomical examination, and introduced a regulatory scheme for schools where anatomical examinations took place.^4^ These statutes supplied bodies for dissection under certain conditions as exceptions to fundamental obligations at common law to effect and preserve a parishioner’s right to ‘Christian’ or ‘decent’ burial and, thus, to facilitate requirements of ecclesiastical or church law.^5^

The common law, in such comments, is associated with the traditional religious feeling of what ought to be done with the dead (namely, Christian or decent burial), a feeling popularly held in England at the time of the *Anatomy Act 1832* and required by ecclesiastical law.^6^ Christian eschatology placed importance on burial so as to preserve the dignity of the body until resurrection at the Last Judgment, and whilst religious elites could tolerate other uses, such as dissection, burial of the body remained integral to ecclesiastical law as that was practised in England.^7^ Common law courts generally deferred to ecclesiastical administration and courts to handle disputes relating to the disposal of the dead,^8^ following the comment of Edward Coke that the dead body was of ‘ecclesiastical cognizance’.^9^ Indeed, Coke suggested that this was why no property could exist in a dead body at common law, which William Blackstone appeared to concur with whilst relying on *Haynes’s Case* (1614) as authority.^10^ But in the eighteenth and nineteenth centuries, common law courts increasingly clarified obligations they could enforce to facilitate ecclesiastical law.^11^ This included requiring the performance of burial from certain persons upon whom the duty to bury was placed,^12^ as well as establishing criminal offences for preventing the decent burial of the dead^13^ or disinterring the buried dead without lawful excuse.^14^

Legislation, in contrast, was responsible for deviations from the tradition held by the common law, deviations tolerated owing to the putative utility of modern medicine and surgery. Anatomy and dissection were thereby authorized only according to the provisions of these statutes; such acts were not otherwise lawful. That image of the law appears to have led some historians to refer to dissection outside the permissions of the *Anatomy Act 1832* as illegal, for want of authority.^15^ Likewise, some jurists, relying on such histories, construct decent burial (and, more recently, cremation)^16^ as all the common law ordinarily permits; most other uses, like anatomy and dissection (outside coronial examinations), depend entirely upon legislation for their authority.^17^ However, the historical record is more complicated than this image of the law. There were jurists who insisted that what could be done at common law was more than decent interment.^18^ Rather, the common law, by their argument, appeared to admit the existence of a power to dissect, claimed by physicians, surgeons, and apothecaries prior to and outside of legislation.

As this article shows, to these jurists, such a power authorized and regulated dissection outside the contexts contemplated by anatomy legislation. This is important to show for at least two reasons. First, a common law power to dissect challenges the narratives ordinarily told about the history of anatomy law specifically, and the law of the dead generally, which is instructive for understanding medico-legal history in England and Wales and throughout the British Empire (where legislation could be scant, such as in the Province of Canada and Nova Scotia).^19^ Secondly, a common law power to dissect may be extant where legislation has not displaced it, and thereby authorize certain uses of the human dead, such as dissection specifically, as well as wider uses if the power is understood to also support a more general principle that the common law did not require burial. The presumptive legality of the body’s use begins analysis at a different point, potentially allowing for a wider array of uses if not dependent on statute. That will be relevant to courts that must adjudicate ‘exceptional’ uses of the human dead or tissue and look to the historical record for guidance, such as when Justice Jackson of the High Court of Justice in England and Wales considered ‘the old authorities on the unlawful treatment of dead bodies’ with respect of the novel practice of cryonics.^20^ Likewise, legislators and others interested in law reform affecting the disposal of the dead and the use of human bodily materials will benefit from a corrected historical record.

As Margaret Brazier has said alone and with Jonathan Montgomery, medico-legal history assists the lawyer in examining the relationships of law and morality to medical practice, by tracing the conditions for their emergence.^21^ Without history, ‘we waste time and effort and repeat the same mistakes’ and ‘[i]f we have only a vague notion of history, a notion unsupported by evidence, we may make bad laws today’.^22^ I specifically engage in medico-legal history as that services doctrinal understandings of medical law, ‘us[ing] formal legal materials such as reports of decisions […] and evidence of lawyers’ argumentation and reflections, in order to reconstruct the mind of the professional legal collective as it understands its own activity in guiding conduct and resolving disputes’.^23^ Re-examining old doctrines can disrupt the orthodoxy of current legal doctrine, suggesting alternative possibilities for law through its retelling.^24^ Especially with the young discipline of medical law, in search of its history and the history of its subject matter,^25^ histories of legal doctrine remain worthwhile.

The article focuses on identifying the historical sources and elements of a common law power to dissect, as expressed by some jurists. Analysis of its broader significance, historically and to contemporary legal doctrine, merits more space than allowed here. But I do occasionally place the power to dissect in the context of bodysnatching, and the inadequate supply of bodies for dissection and anatomical examination, which precipitated the *Anatomy Act 1832*.^26^ Bodysnatching involved disinterring the buried dead or, more dangerously, murder, so to procure bodies for dissection and anatomical examinations.^27^ The practice emerged in response to the inadequate supply of bodies for anatomical examination, especially as private and university anatomy schools were increasingly opened in the late-eighteenth and early-nineteenth centuries, creating greater demand.^28^ It is necessary for me to place the power in this context, as it will assist the reader of legal history in gaining a fuller understanding of the policies underlying the doctrine and its use. Furthermore, the context preceding the *Anatomy Act 1832* also supplied at least two cases that led some from this period to conclude that anatomy and dissection were unlawful at common law.^29^ These cases are important to analyse since they may be raised to negate the existence of a power to dissect.

The article has three parts: first, I show where and how jurists in the common law described the power to dissect; secondly, I set out the power’s elements, particularly as it interacted with the ‘no property’ rule, criminal law, the law of tort, and any other municipal law; and thirdly, I observe how the power to dissect was threatened by concern for bodysnatching but ultimately persisted despite how certain cases were narrated.

## II. THE COMMON LAW POWER

The common law power to dissect was suggested in at least three cases: in the *obiter dicta* of the Court of Queen’s Bench in *R v Price* (1884), *dicta* of the Québec Superior Court (as it was then known) in *Phillips v Montreal General Hospital* (1908), and in the reasons of an inquiry in *Re Ramsay Smith* (1903).^30^ It was also referenced in manuals (on the law of the dead and hospital law) written in the late-nineteenth and early-twentieth centuries.^31^ These sources evidence a common law power to dissect exercised by those trained in medical arts.^32^

The argument was given its most authoritative statement in *Price* (1884). *Price* (1884) was about the legality of cremation at common law, but Justice Stephen of the Queen’s Bench also commented on anatomy.^33^ In doing so, he wrote critically of the claim that burial was all the common law admitted.^34^ He made two claims: firstly, the common law established duties on individuals to effect a decent burial (and later cremation), but that did not mean it occupied the field as the only option.^35^ Cases that addressed the obligation to bury the dead dealt with situations where the question of burial, as the predominant course of conduct at the end of life, was necessarily engaged;^36^ those decisions often referred to a duty to bury, but Justice Stephen felt it was unsupported by reason to claim that these cases stood for more than their narrow questions and established an ‘absolute’ obligation to bury the dead.^37^ As Justice Stephen noted, to take the ‘language of the cases referred to […] as if it were intended to be severely and literally accurate’ would bar legitimate activities like the lawful access to, and the trade of, ‘skeletons and anatomical preparations’.^38^

Secondly, Justice Stephen referred to the legality of anatomy at common law as he reasoned through the application of past authorities to unusual or exceptional uses of the dead. Anatomy, like cremation, was ‘an exceptional method of dealing with dead bodies’, but despite its exceptionality, Justice Stephen observed that it went ‘[without] interference on the part of the [British] legislature down to the year 1832’ when the UK’s *Anatomy Act 1832* was enacted.^39^ From the history of anatomy in England, and the language of the *Anatomy Act 1832*, he inferred ‘that Parliament regarded anatomy as a legal practice [when it introduced the *Act*], and further, that it considered that there was such a thing as “a legal supply of human bodies,” though that supply was insufficient for the purpose’.^40^ Having regard to this context, Justice Stephen concluded that anatomy was a lawful practice at common law,^41^ writing ‘that “[t]he practice of anatomy is lawful and useful though it may involve an unusual means of disposing of dead bodies, and though it certainly shocks the feelings of many persons”’.^42^ The exceptional or alternative use of the human body was subject only to requirements of criminal law (against indecency or public nuisance, for example), tort law (in an action for nuisance), or some other municipal law.^43^

The Court’s comments were properly *dicta*—the complaint under adjudication pertained to the legality of cremation and not anatomy—but analogies to anatomy were persuasive, and some law writers (at least until the mid-twentieth century) appear to have agreed.^44^ The law writer E. Lewis Thomas, for example, in his 1901 edition of *Baker’s Law Relating to Burials*, stated that ‘[t]he custom of disposing of the dead in this country […] has from time immemorial been by burial, but it cannot now be doubted that disposal by other decent means is lawful’. Lewis Thomas cited *Price* (1884) as authority for this general proposition (‘other decent means’ are lawful), from which he paraphrased Justice Stephen’s analysis to demonstrate:

many dicta [were] to be found in previous judgments apparently treating burial as the only legal way of disposing of the dead bodies, [but these] were not to be regarded as declarations of the illegality of cremation, but were to be explained as having been used in cases where no question as to cremation arose.^45^

When discussing *Price* (1884), Lewis Thomas referred only to the Court’s decision regarding cremation, but its reference as authority for the general proposition (‘other decent means’ are lawful) over the narrow proposition (cremation is lawful) suggests he agreed with the Court’s comments on anatomy.^46^ Likewise, James Brooke Little, in his 1894 edition of *The Law of Burial*, wrote:

In many cases which have come before the courts, expressions have been used which imply that no method of disposing of the dead body other than by Christian burial (with certain ecclesiastical exceptions) is lawful in this country; but these cases were all examined by STEPHEN J, in his charge to the jury in *Reg v Price*, and that learned judge expressed his opinion that those expressions must not be taken as deciding that no other method of disposing of the dead is lawful, and that the courts never intended to lay down any such rule. Where such expressions occur, therefore, they should be taken as indicating that a duty rested upon somebody of decently disposing of the dead body in question, but that such duty might be discharged either by burying it or disposing of it in some other lawful manner, such as by burning.^47^

Here, too, cremation was merely an example of ‘disposing of [the dead] in some other lawful manner’. Brooke Little otherwise claimed a general proposition that could capture anatomy as discussed in *Price* (1884).

Among law writers, agreement with Justice Stephen was most explicit in the comments of Arthur Turnour Murray. In his 1908 text, *The Law of Hospitals*, Turnour Murray quoted from *Price* (1884) under the heading, ‘Dissection at Common Law’,^48^ stating that ‘[t]he practice of anatomy is lawful and useful, though it may involve an unusual means of disposing of dead bodies and though it certainly shocks the feelings of many persons’.^49^ Further, he added that ‘[p]rior to the Anatomy Acts […] this practice [of dissection] continued unchecked’, although the incidents of bodysnatching were regulated by criminal law as misdemeanours.^50^ Turnour Murray appears to have thought the common law power to dissect was restricted by the *Anatomy Act 1832*, as he otherwise did not discuss the common law power; instead, he proceeded to discuss dissection with respect to the *Anatomy Acts*, even though the *Anatomy Acts* only referred to anatomical examination (a distinction that some made, such as in *Re Ramsay Smith* (1903)).^51^ But Turnour Murray’s reference to dissection at common law and his quotation from *Price* (1884) show how Justice Stephen’s opinion could be read favourably to the existence of such a power.

Justice Stephen’s opinion was also shared (although not cited) by other cases, including a decision of the Québec Superior Court, which suggested that dissection could be lawful even without explicit statutory authorization.^52^ In *Phillips v Montreal General Hospital* (1908), the Québec Superior Court considered whether a widow could bring an action for mental suffering caused by a physician’s dissection of her late husband without her consent. The Court did not comment on the case’s merits, but it concluded that the widow could bring such an action, as it was arguable that she had a possessory interest in the remains (more on that below). But in reaching that decision, the Court also commented on the legality of alternative uses of the dead. The Court wrote that ‘blind acceptance of [the] doctrines [of no property] would lead to the conclusion, from every conceivable point of view, that a cadaver is, as the word implies, only flesh given to the worms and in regard to burial nullius in bonis’;^53^ such a conclusion dissatisfied, in that it obstructed utilities for the living derived from the dead and their parts, such as with ‘skeletons and anatomical preparations of parts of dead bodies’ (of relevance to anatomists), and disregarded the interests people form in relation to such matters (of relevance to widows).^54^ Further, whilst the ‘non-development of English case law’ on human remains and tissues ‘may be to some extent accounted for by the separate authority which English ecclesiastical courts exercise[d] over the custody, burial and exhumation of the dead’,^55^ the Court referred to the *Anatomy Act 1832* as ‘giv[ing] emphasis to the belief that something more than a mere duty of sepulture exists in respect of a dead body’.^56^ Although only a decision on demurrer, holding that the circumstances disclosed a cause of action, the Court made plain that they saw the English common law as potentially allowing for more than final disposal, to the extent that the use was not otherwise unlawful or a criminal offence.^57^

Likewise, an inquiry into charges against Dr William Ramsay Smith (1903)—a notable medico-legal authority in Australia who held various public appointments in the Colony of South Australia and the Dominion of Australia in the late-nineteenth and early-twentieth centuries^58^—convened by the Legislative Assembly of South Australia concluded that Ramsay Smith was authorized by the common law (‘the law of the land’) to dissect remains in his possession or with the consent of those who had lawful possession.^59^ The charges related to misconduct alleged to have occurred during the performance of his duties as city coroner, among other public offices; namely, gruesome mutilations of bodies of deceased humans, creating specimens of soft tissue, skeletons, bone fragments, etc,^60^ which in many cases were sent overseas to the University of Edinburgh.^61^ Importantly, none of these dissections were with the authority of anatomy legislation, nor were they authorized by the office of coroner; yet, the Board of Inquiry concluded that it did not matter.^62^ Ramsay Smith was entitled to do as he did at common law, a power that existed for duly qualified medical men ‘before [any anatomy] act was passed’.^63^

The Board offered their interpretation of the laws pertaining to the use and disposal of dead bodies and body parts, noting the ‘absence of judicial authority’^64^ on the precise questions raised regarding these charges (which alleged breaches of South Australia’s *Anatomy Act 1884*).^65^ The Board provided a full-throated defence of the claim of a common law entitlement to dissect, concluding that the absence of authority under the *Anatomy Act 1884* was inconsequential:

The question of legality or illegality of the matters charged, for the most part, depends upon what is held to be the true scope and effect of the “Anatomy Act of 1884” (No. 317), and, in the absence of judicial authority, we deem it necessary to shortly state our views of the effect of that Act. The charges referred to, so far as they relate to breaches of the Anatomy Act, are that Dr Ramsay Smith, not being a person licensed under that Act, performed anatomical examinations at places not licensed under the Act, and that such examinations were, therefore, illegal; and sections 4, 12, 14, 16, and 18 were particularly relied upon.

We are of the opinion that this is a mistaken view of the law. The Act, in its main provisions, is similar to the Imperial Anatomy Act (2&3 of Wm. IV., c. 75). Before that Act was passed, it appears to us that anatomical examinations were lawfully taught and openly practised. The whole history of the subject shows that anatomy of the human body developed from most ancient times to a systematic practice, resulting in the establishment of schools and other institutions for that purpose.^66^ (my emphasis)

Furthermore, the Board noted bodies were lawfully dissected after being obtained from those who had lawful possession, which could be the next of kin or others, such as hospitals and physicians:

So general was the practice of anatomy [since the 17^th^ century] that the legitimate demand for dead bodies for anatomical dissection brought into existence the class of wretched criminals known as “Resurrectionists,” and even murderers, such as Burke, who was executed for the offence in 1829. We differ with the contention of [counsel for the Attorney General], that at that time the only legal source of supply of bodies for anatomical dissection were those upon which *post-mortems* had been held under coroners’ inquests and the bodies of murderers under the statute law. It appears obvious from the history of anatomy that another, and the chief source of supply, was the bodies obtained from relatives or other persons having the lawful possession. […] It cannot be doubted that, if it had been unlawful to practice on bodies supplied by the persons in lawful possession, the reports would have contained many cases of prosecutions. That, in our opinion, was the state of the law up to the passing of the Imperial Anatomy Act in 1832.^67^ (my emphasis)

That state of the law continued in England despite the *Anatomy Act 1832* and the *Anatomy Act 1884* in South Australia, having regard to the historical context, the language of the acts, and the titles thereof (‘to authorise the establishment of Schools of Anatomy, and to regulate the practice of anatomy therein’):

[The *Anatomy Act 1832*] was a complete recognition of the existence of a proper demand, in the interest of the living, of bodies for dissection, and it increased the supply, particularly from certain public institutions. Our Act makes it a condition that the bodies to be dissected under its provisions go only to Anatomical Schools, which were to be licensed, and that the bodies themselves should be anatomically examined by students and others, all of whom were also to be licensed and be subject to the control of inspectors. Each of the Acts was a measure to facilitate and regulate the systematic study and practice of anatomy, and not a measure to curtail or interfere with the right of a medical man to perform surgical operations,  *post-mortem*  examinations, and removal of parts of the body, as in his judgment might be necessary; all of which remained, in our opinion, unaffected by the Act. It is his business to do all such things. He is paid for his judgment and skill for the purpose, and his very living will always depend upon his discretion and nice sense of what is fitting between himself and his patients and their friends. Each medical man must judge for himself, and at his own risk, what is the proper course of action in each particular case.^68^ (my emphasis)

The Board thereby asserted that physicians—whether in private practice or at public institutions—were ‘equally free to follow the ordinary rules and usages of the medical profession as to performing *post-mortem* examinations, where deemed necessary and in proper circumstances, and by so doing, no breach is committed of the provisions of the Anatomy Act’.^69^ The Board held that ‘it is certainly a part of the duty and in the ordinary course of the practice of the medical profession, to remove parts of the human body from the living as well as from the dead bodies’, and that the amount of human matter to be removed ‘and how it should be disposed of, must of necessity rest on the judgment and good taste of the medical man in the particular case’.^70^ There was also no evidence that Ramsay Smith acted against the wishes of individuals in lawful possession of the bodies*, if not him*.^71^ Accordingly, Ramsay Smith had not committed any offences with which he was charged in taking custody, dissecting, retaining, or transferring bodies and parts.^72^ Exoneration was not provided by way of legislative permission; rather, the Board drew on language that strongly characterized a right at common law. In this way, the Board recognized the lawfulness of what was described by numerous witnesses as being ‘usual practice’,^73^ without ‘impropriety’,^74^ and ‘regarded as custom’.^75^

## III. ELEMENTS OF THE POWER

Most sources did not describe what the power entailed, except to imply that someone trained in medical arts was authorized to dissect human remains. It was only in the reasons of the inquiry in *Re Ramsay Smith* (1903) that elements of the power were given.

The inquiry in *Re Ramsay Smith* (1903) found that medical men like Dr Ramsay Smith could dissect human bodies of patients who died in their charge and whose bodies were thereby in their lawful possession, or with the consent of whoever had lawful possession of the bodies (they believed this would ordinarily be other medical men or potentially institutions, like hospitals).^76^ Dissection was allowed where in the medical man’s judgement it was necessary to advance their learning and scientific study, perhaps owing to an irregularity of disease or injury, or the tissue having some other special quality (for Dr Ramsay Smith, that included the racialized differences attributed to Indigenous peoples in Australia). Where the next of kin claimed interests in the bodies of the deceased and objected to dissection, customarily medical men would not dissect unless it could be concealed and done without causing offence. Anatomical specimens could also be prepared from bodies that next of kin claimed, irrespective of their objections, if done clandestinely. In all circumstances, dissections were to be carefully done, undertaken with the propriety of their office as medical men, without wanton disregard or mutilation.

Dissection was lawful in this way, as opposed to merely not being unlawful, owing to professional privileges. The laws and customs of the physician, as a professional office bestowed on Dr Ramsay Smith, authorized him to act as he did. That same office thereby structured how he carried out those dissections, in accordance with their laws and customs. That office-based lawfulness—in the sense of being part of an institution which ‘mark[s] the duties, responsibilities, rights and privileges that are taken up in public life’^77^—was carried by physicians, surgeons, and apothecaries throughout the British Empire, as well as the sanction of their office at common law, without dependence on colonial legislation.

### A. Relationship of the power to the ‘no property’ rule

Other elements must be inferred from the situation in which the claim likely found expression and the laws likely to have interfaced with the power. An important, enabling condition to the power to dissect was the common law rule that the corpse, and parts thereof, were *nullius in bonis* (belonging to no one) and generally incapable of being property.^78^ Defenders of Dr John Scott of Toronto in 1851, for example, made the claim that Dr Scott was entitled to dissect because the remains of his patient were *nullius in bonis*, and so no one had better claim to them than him.^79^ Relatedly, some of those who testified before the inquiry in *Re Ramsay Smith* (1903) observed that the corpses at issue were not property, and so Dr Ramsay Smith was insulated from the claims of next of kin.^80^ There was a quality to the relation asserted by the no-property rule, which simultaneously denied property to others whilst assuring the surgeon, physician, or apothecary’s use of that in their possession.

An early case which supported that conclusion was *Exelby v Handyside* (1749), heard by the Court of Common Pleas at Westminster Hall.^81^ A man (Mr Exelby) brought an action under the tort of trover against an ‘eminent man-midwife’ (Dr Handyside), who delivered the twin births of Exelby’s wife. Exelby argued that Handyside wronged him by taking the bodies of his two conjoined children, who were either stillborn or died shortly after birth;^82^ if the action was good, trover would have entitled Exelby to the value of the chattel (eg, the corpses) converted by Handyside’s unlawful interference. The matter was settled in favour of Exelby with Handyside returning the corpses to him before the jury could reach a verdict (perhaps Handyside agreed out of concern for his reputation, given the unwanted attention he might have received at the crowded Westminster Hall and the nature of his relations—a Dr Handyside, physician of Red Lion Square, married a near relation of Lord Castlehaven earlier that year) but,^83^ at some point during the long trial at Westminster Hall,^84^ Chief Justice John Willes of the Court of Common Pleas reportedly said an action ‘would not lie, as no person had any property in corpses’.^85^ Chief Justice Willes’ comment favoured Handyside and his fellow medical men, and it apparently made an impression despite the case settling.

There are no surviving records of Chief Justice Willes’ comment contemporaneous with it being said,^86^ but the comment was later passed onto the jurist Sir Edward Hyde East, who referred to the case in his *Treatise of the Pleas of the Crown*, published in 1803. Hyde East cited *Exelby v Handyside* (1749) as authority for the no-property rule, and his summary of the case has sometimes been cited for such authority—authority that Paul Matthews says should be impugned, given that Chief Justice Willes’ reported remarks could only ever be dicta to modern sensibilities.^87^ Irrespective of the case’s value as precedent, Chief Justice Willes’ comment is evidence of historical understanding of how the no-property rule took effect. It shows that the rule could be beneficial to those trained in medical arts, as the rule could negate claims of interest in those surviving the deceased. Belonging to no one in common law, the corpse effectively became the property of those proximate to and in possession of it. Medical men thereby obtained a power to dissect over what no one else could claim,^88^ although, as suggested above, the power could not be exercised wantonly—there were elements to its proper use derived from its original authority as medical privilege.^89^ The beneficial relationship between the power to dissect and the no-property rule reflected what Remigius Nwabueze calls the no-property rule’s duplex character—it indexes a complex relation that simultaneously denudes some of power and appropriates power for oneself.^90^

But the power to dissect did not require the no-property rule’s duplex. Even in those (so far) rare instances where courts recognized the dead body as the property of the next of kin, or at least as something in which near relations had some interest, that did not necessarily deny the possibility of there being a power to dissect. Rather, in those situations, the power became conditional on the authorization of the next of kin or near relation (unless dissection formed part of the coroner’s inquest, where no consent was required).^91^ The lawfulness of dissection remained intact; merely, more people were involved in the decision to exercise the power. For example, the power’s pliability is suggested in the Canadian cases of *Phillips v Montreal General Hospital* (1908), discussed above, and *Edmonds v Armstrong Funeral Home Ltd* (1930), decided by the Alberta Court of Appeal, as well as the English case of *R v Redmond* (1818), decided by a magistrate’s court at Guildhall.^92^

In *Phillips* (1908), the widow complained that she had not authorized the dissection of her late husband and brought an action against the hospital for damages in mental suffering. The Québec Superior Court held that the widow could bring an action, as it was arguable she had a possessory interest in the remains of her husband, which was injured as a result of the dissection. Combined with the Court’s comments on alternative uses of the dead, including the preparation of specimens and skeletons, the Court seems open to dissection being lawful where dissection did not interfere with the possessory interests of the next of kin.

In *Edmonds* (1930), the Alberta Court of Appeal decided that the next of kin’s duty to decently dispose the remains of their relation included ‘the right to the custody and control of the remains […] [and] any unauthorized interference with that right, such as [dissection without the next of kin’s consent], was an invasion of his right and would give rise to a cause of action’.^93^ The Court observed that their case ‘was exactly like’ *Phillips* (1908),^94^ in that a doctor had performed an examination of the plaintiff’s relation and removed portions of the body without the plaintiff’s consent.^95^ Here, the plaintiff had begun arrangements for his wife’s burial—her body was at the funeral home when the doctor carried out his dissection—which seemed to reinforce for the Court that the plaintiff’s authorization was necessary for dissection to occur.

Finally, in *Redmond* (1818), the magistrate’s court at the Justice Room of London’s Guildhall heard a dispute between Dr Chumley and Mary Redmond, the mother of an infant patient who was in his care.^96^ Dr Chumley brought a charge against Redmond who ‘threatened to destroy him, and [whose] words […] increased the Doctor’s apprehensions for his safety in no ordinary degree’.^97^ Redmond’s child was admitted to Guy’s Hospital, but the child died whilst Chumley was out of town, and a dissection was carried out by his colleague without Redmond’s consent; the dissection was reportedly conducted in accordance with ‘a privilege existing in the Hospital to which he belonged, as well as in all others, which authorised medical men, in case anything uncommon attended the decease of a patient to open the body, in order to ascertain the cause’.^98^ The body of the child was returned to Redmond ‘mangled’, with ‘the throat cut, and the bowels taken out and half-stitched up’.^99^ It was at this point, Redmond began requesting the return of dues paid, threatening to ‘rip up [Chumley’s] bowels and cut his throat’ and ‘calling crowds round [sic] his house and the hospital’ to alert them to her cause.^100^ The alderman presiding over the court was reportedly critical of Chumley, saying that ‘there existed a feeling in all parents in cases of the kind, and whatever necessity might have existed [to carry out the dissection], the body should not have been opened without consulting the mother’.^101^ Further, he repeated ‘that without the consent of the parent the thing should not have been done’, and that ‘[t]here was no justification for it [dissection without the mother’s consent]’.^102^ Chumley’s prosecution of Redmond failed owing to the ‘outrage’ caused to Redmond, and the alderman discharged Redmond from bail with only a warning not to follow Chumley for money.^103^ Here, the act of dissection was not impugned, but the alderman required dissection to be conditional to the parent’s consent.

The involvement of kin in the authorization of dissection thereby modified, rather than squelched, the power to dissect in these situations. Whether justified on theories of property, as in *Phillips* (1908), or on sentiment or duty, as in *Redmond* (1818) or *Edmonds* (1930), interests could extend to the surviving next of kin, which factored in whether dissection could be undertaken.^104^

### B. Shape given by criminal, tort, and municipal laws

Irrespective of what the laws of surgeons, physicians, and apothecaries might have theoretically allowed, their actual expression appears to have been fettered by the jurisdictions interfacing with them; namely, criminal law and the law of tort, as well as any other municipal law.^105^ Such laws gave the power its proper shape, forming the outer envelope of what could be done with the human dead and parts thereof.

*Price* (1884) is again perhaps the best authority for this statement. Justice Stephen held that the exceptional use of the human body (such as cremation or dissection) was subject only to requirements of criminal law (against indecency or public nuisance, eg), tort law (in an action for nuisance), or some other municipal law.^106^ With respect to criminal law, Justice Stephen observed that, in order for him to have declared burning a dead body was a misdemeanour for indecency, he had to be satisfied ‘not only that some people, or even that many people object[ed] to the practice, but that it [was], on plain, undeniable grounds highly mischievous or grossly scandalous’.^107^ This was a power he had to exercise cautiously, noting that, at common law at the time, ‘many acts involving the grossest indecency and grave public mischief […] [were] not misdemeanours’.^108^ Whilst the historical ‘disuse of burning bodies’ and embrace of burial ‘was due to the force of [religious] sentiments’ that defined English society, Justice Stephen did not believe it ‘[could] be said that every practice which startle[d] and jar[red] upon the religious sentiments of the majority of the population [was] for that reason a misdemeanor at common law’.^109^ Accordingly, as long as the dead body was burned ‘decently and inoffensively’ it was ‘not criminal’. But, he added, ‘if it [was] done in such a manner as to be offensive to others it [was] a nuisance of an aggravated kind’. Such a nuisance was an act which ‘obstruct[ed] or cause[d] inconvenience or damage to the public in the exercise of rights common to all Her Majesty’s subjects’, and that might have been the case in *Price* (1884) had the jury determined there was evidence the body was burned in ‘such a place and such a manner as to annoy persons passing along public roads or other places where they have a right to go’.^110^ Given Justice Stephen’s earlier analogy to dissection and anatomy, a similar analysis would apply to these uses as well.

The application of public nuisance to dissection is exemplified in *Vade’s Case* (1818).^111^ Mr Vade was summoned to the City of London’s Mansion House, where the Lord Mayor of London presided over a dispute between Vade and his neighbours. Vade was a chemist and a student of surgery, and he had some of his dissection subjects at his home hung on top of the roof. Vade’s neighbours detected a horrid smell and discovered its putative source in the human viscera exposed and strewn across his roof. The neighbours then complained that Vade had caused a nuisance to them. The Lord Mayor, after hearing evidence, determined there was a nuisance and ordered Vade to remove the source of it. If Vade did not remove the human viscera, the Lord Mayor threatened to direct the City Solicitor to bring an indictment against Vade for the nuisance (in this way, it appears the Lord Mayor thought Vade committed a public nuisance as opposed to a private nuisance). Vade agreed to remove the nuisance, although the newspaper stated he did so hesitatingly. The Lord Mayor was reported to have said that he ‘was aware that the dissection of dead bodies was necessary; but then the operation should be conducted with a proper regard to decency, and due care should be taken not to annoy the Public [sic]’ (again suggesting this was a public nuisance).^112^ The Lord Mayor’s order thereby only related to the injuries caused by the smell of viscera to others, and not to the dissection itself. From this, the Lord Mayor appears to have thought dissection was lawful even if done within a private dwelling and without the authorization of legislation, with the tort and offence of nuisance partly shaping the outer envelope of dissection’s lawful exercise.

Municipal law generally could also regulate the power to dissect. For example, section 161 of the UK’s *Public Health Act 1936* and section 296 of the *Public Health (London) Act 1936* authorized the Minister of Health to impose conditions or restrictions on the ‘means of disposal [of human remains] otherwise than by burial or cremation’ to protect ‘the interests of public health or public safety’,^113^ suggesting that exceptional uses of the human body, like the power to dissect, were contemplated and regulated. Law writer, Alfred Fellows, referred to these sections to show that uses other than burial and cremation were possible and that, whilst such uses were ordinarily subject to tort and criminal law, they were sometimes subject to legislation.^114^ Section 142 of the UK’s *Public Health Act 1875* also granted permission to ‘any justice’ to ‘order […] the removal’ of a body to a mortuary ‘[w]here the body [was] of one who [had] died of any infectious disease’ and ‘retained in a room in which persons live[d] or [slept]’, or where the body was ‘in such a state as to endanger the health of the inmates of the same house or room’.^115^ Such authority could theoretically interface with the power to dissect, as Fellows’ gloss suggests,^116^ helping determine what could be lawfully done.

Invoking the power in situations squarely regulated by the *Anatomy Act 1832*, especially to justify contraventions of the *Act*, could also make the dissection unlawful. For example, in *R v Parrott* (1855), a surgeon was convicted of a misdemeanour at the Surrey Quarter Sessions after he obtained and dissected a deceased workhouse inmate outside the circumstances of the *Anatomy Act 1832*.^117^ Dr Parrott claimed to have been a friend of the deceased, who examined him at his friend’s final request; but that meant obtaining the body from the workhouse by pretending he had the authority of the nearest relatives.^118^ Dr Parrott claimed that he dissected the deceased’s body as he was entitled to as a surgeon—such examinations were ‘an everyday occurrence’^119^—but that was no defence to his failure to notify the Anatomy Inspector of the intent to remove the deceased from the workhouse and he was convicted for failing to comply with the ‘form of the statute’.^120^ But dissections completed by Dr Tannahill at a hospital in Glasgow, without notice to the Anatomy Inspector, might not have contravened the *Anatomy Act 1832*; in the case of *Re Stevenson and Others* (1877), the prosecutor stopped pursuing the charge against Drs Tannahill and Stevenson after an advocate appearing for Dr Tannahill argued that such dissections were examples of *post-mortem* examinations done by a competent legal authority (ie Dr Tannahill) and, relying on section 15 of the *Anatomy Act 1832*, that the *Act* ‘took no cognisance of post-mortem examinations’.^121^ Implicit to the argument that Dr Tannahill was the competent legal authority is that he had a right to dissect outside the *Anatomy Act 1832*; indeed, as his advocate argued, his right to dissect was a necessary precondition to any certificate stating a cause of death, which the *Act* otherwise required to be delivered with notice to the Anatomy Inspector if the body were to be transported to a licensed school for anatomical examination.^122^ Further study is needed on the interaction between common law power and anatomy legislation, and the conflation of *post-mortem* examinations and dissection, but these cases suggest that there is something in the situation of the dissection and its resemblance to the customs of medical practitioners in determining whether contraventions occurred.

## IV. THE THREAT OF BODYSNATCHING

Challenges to common law power appeared in the early-nineteenth century. The cases and comments I have surveyed were largely written in the late-nineteenth and early-twentieth centuries, when popular feeling about dissection had perhaps improved. But in the late-eighteenth and early-nineteenth centuries, popular feeling in England was stirred by bodysnatchers. That popular feeling seemed to reinforce an older antagonism to dissection, frequently expressed by the eighteenth century by riot, preventing the Barber-Surgeons from taking the bodies of executed felons.^123^ Dissection violated popular religious belief by preventing the body from reunifying with the soul in heaven, and so surgeons became the object of public unease and violence.^124^ In that context, dissection occupied a precarious position in England, with Barber-Surgeons petitioning the King for protection as they retrieved bodies from the gallows.^125^ Magistrates also increasingly presided over trials of bodysnatchers and sometimes their medical associates,^126^ often enthusiastically followed by the public,^127^ which suggested, to some, that the common law penalized the mere possession of a dead body unless explicitly provided for by statute (eg the *Murder Act 1751*).^128^

The UK’s Select Committee on Anatomy in 1828—a parliamentary body convened for the purpose of studying the state of anatomy law and the need for legislation—was one of those concerned with the state of the common law.^129^ The Committee’s proceedings show that members and some witnesses frequently referred to two cases—cases of *R v Wilkinson and Young* (1785) and *R v Davies and Another* (1828)—with urgency.^130^ Although both cases were unreported, in the sense that the reasons for the courts’ decisions were not published, witnesses were asked about the cases, which generally elicited the opinion that legislation was necessary to rid the problem of bodysnatching and to ensure dissection’s lawfulness.^131^ In the final report, the Committee wrote that:

At present [in 1828], however, a most intelligent magistrate, [Samuel Twyford of Bow-Street] one of the witnesses, […] infers from the analogy of all these cases, that to treat a dead body as liable to any thing but funeral rites, is an offence *contra bonos mores*, and therefore a misdemeanour.^132^

Accordingly, although ‘magistrates appear[ed] hitherto to have taken no cognizance of receiving into possession a dead body, unless there was strict evidence that the receiver was a party to the disinterment’,^133^ the Committee enjoined the House of Commons to pass legislation to legalize another supply of bodies to anatomy schools, rid the UK of bodysnatchers, and clarify the authority to practise anatomy and dissection. These events cast doubt on the validity of a common law power to dissect.

However, *Wilkinson and Young* (1785) and *Davies* (1828) do not appear to stand for the rule that anything but decent interment was a misdemeanour. Reliance on *Wilkinson and Young* (1785) was especially dubious. Having found and read the relevant indictments and crown rolls of the King’s Bench in 1784 and 1785, respectively, it does not appear that Lord Mansfield held dissection was illegal.^134^ Rather, the accused—Robert Wilkinson (master of the workhouse) and Thomas Young (surgeon)—were found guilty of misdemeanours for preventing, and the conspiracy to prevent, the decent burial of human remains of workhouse inmates, which was both required by the ‘Law and Customs of the Realm’ and Wilkinson’s duty, as the master of a workhouse, to so dispose.^135^ The indictments averred to dissection, but always in conjunction with conspiracy to prevent burial and procure the bodies for something other than burial, and with emphasis on the bodies being of dependents of workhouses.^136^ The misdemeanour, then, was not Young dissecting the bodies, but Wilkinson’s failure as workhouse master to provide for their burial and Young’s involvement in that conspiracy.

Similarly, the court at the Lancaster Assizes did not find dissection illegal in *Davies* (1828), although Justice Hullock reportedly called the dissection ‘unlawful’.^137^ An extract shows that the case concerned a conspiracy to disinter a body for the purpose of procuring it for dissection, in addition to charges of having ‘unlawfully procure[d], and receive[d], and [took] into their possession the body’ that was ‘unlawfully and indecently disinterred’.^138^ All the defendants were acquitted of charges pertaining to the conspiracy—the jury was presumably not satisfied, as Justice Hullock instructed, ‘that the conduct of the defendants was the result of previous concert’.^139^ But John Davies (a medical student) and William Blundell (an accomplice) were convicted of the other charges relating to the possession of the body unlawfully and indecently disinterred—and two of those charges referred to ‘the intent that the same [body] should be unlawfully dissected’.^140^

The Select Committee was principally concerned with the comments to the jury, which suggested that there was such a thing as ‘unlawful dissect[ion]’ and that ‘[t]he only bodies legally liable to dissection […] were those of persons executed for murder’.^141^ But the comments about dissection were unnecessary. It was the unlawful disinterment of the body—disinterment without licence or some other lawful excuse—which was the misdemeanour; the fact that the parties intended the body to be dissected was inconsequential. Otherwise, it would have been unnecessary for the jury to be satisfied that there was clear evidence that Dr Hall—a surgeon who sourced and provided monies for the disinterred body, and was one of the accused—had knowledge of the body’s unlawful disinterment;^142^ knowledge that the body would be used for dissection would have been sufficient for a conviction. Furthermore, Dr Moss—a physician who consented to the reception of the body in his house for the purposes of dissection, but not included in the indictment—would have been vulnerable to criminal action. Rather, the offence was predicated on the existence of unlawful disinterment which, as Justice Bayley said at sentencing of Davies and Blundell, ‘distress[ed] the feelings of the surviving friends of persons whose bodies were thus disinterred’.^143^

Accordingly, the principles upon which Lord Mansfield relied in *Wilkinson and Young* (1785), and Justice Hullock in *Davies* (1828), do not appear to reflect meaningful expansions of criminal law. Rather, these cases were consistent with *Lynn* (1788) and other cases concerned with preserving the decency of burial once undertaken.^144^ Nonetheless, rediscovery of *Wilkinson and Young* (1785), and reports of *Davies* (1828), shook the confidence of the Select Committee especially when those cases were glossed in the testimony of an eminent magistrate. The cases portended greater risk of liability, especially as the imaginations of the public were animated with bodysnatchers. Altogether a feeling developed that ‘the state of the law’ was ‘injurious’ and caused a particular ‘hardship’, and that this required change, otherwise the anatomist’s position was untenable.^145^

The *Anatomy Act 1832* would eventually pass, and public feeling calmed.^146^ That seemed to stop discussions of dissection being a misdemeanour at common law, perhaps because the authority to dissect was less closely scrutinized by the public, even though medically trained persons continued to engage in dissection outside the circumstances of the *Anatomy Act 1832*.^147^ Altogether, shifts in sentiment in the mid- to late-nineteenth century appeared to create the occasion for cases and comments favourable to the common law power to dissect, free of the memory of bodysnatching.

## V. CONCLUSION: DISSECTING MYTHS

The origin of the common law power to dissect is occasionally located in ancient customs, which preceded and were not immediately altered by legislation such as the *Anatomy Act 1832*.^148^ The *Anatomy Act 1832* was needed to respond to the insufficient supply of bodies for anatomical schools, the latter of which rapidly increased in number and created a demand for the practice of bodysnatching.^149^ But legislation was not, in their view, necessary for practitioners’ authority to dissect. Otherwise, as a writer in the Lancet observed in 1831, how else did people lawfully bequeath their bodies to practitioners for dissection (for such bequests to be lawful, which they assumed they were, dissection also had to be lawful absent any legislative authorization)?^150^ This was also apparently the view of counsel of the Royal College of Surgeons of England in a statement written in 1829, who wrote, ‘[a]s the law […] at present stands […] it may be doubtful whether there be any authority for saying that dissection is unlawful or that executors should be guilty of a misdemeanour in delivering a body on any member of the profession’.^151^ These were historical uses in England that some proclaimed were protected by the law of the land, including the colonies.^152^

A common law power to dissect challenges the histories told about anatomy law in England and Wales, and the British Empire. Instead of establishing burial as fundamental to the common law, the common law would appear open to other uses of the dead. That revises the stories gleaned from old doctrine and suggests alternative possibilities for medical law around dissection specifically, and the disposal of the dead more generally. Those stories cannot displace moral reflection—medical law need not inherit old doctrines—but they may provoke thoughts about the laws by which we live and die.

## Footnotes

1 See eg, Helen MacDonald, *Possessing the Dead* (Melbourne University Press 2007); Ruth Richardson, *Death, Dissection and the Destitute* (University of Chicago Press 1989).

2 32 Hen VIII c 42 (E&W). A similar act was apparently enacted in 1565 granting four bodies to the College of Physicians of London, and another in 1663 granting six bodies to the Company of Barbers and Surgeons of London, see Alan F Guttmacher, ‘Bootlegging Bodies: A History of Bodysnatching’ (1935) 4 Bulletin of the Society of Medical History of Chicago 353, 357.

3 25 Geo II c 37 (UK).

4 2 and 3 Will IV c 75 (UK).

5 Regarding Christian burial, see *R v Coleridge* (1819), 106 ER 559, 2 B & ALD 804 (King’s Bench) (E&W); *Gilbert v Buzzard and Boyer* (1820), 3 Phill 335, 161 ER 1342 (Consistory Court of London) (E&W). Also see *R v Stewart* (1840), 12 AD&E 773, 113 ER 1007 (Queen’s Bench) (E&W); *R v William Vann* (1851), 2 Den 325, 169 ER 523 (Queen’s Bench) (E&W).

6 Margaret Brazier, ‘The Body in Time’ (2015) 7 Law, Innovation and Technology 161.

7 ibid.

8
*Coleridge* (n 5); *Gilbert* (n 5).

9 Edward Coke, *The Third Part of the Institutes of the Law of England: Concerning High Treason, and Order Pleas of the Crown, and Criminal Classes* (M. Flesher 1644) 203. Also see William Blackstone, *Commentaries on the Laws of England, Book 2* (Clarendon Press 1768) 429; William Blackstone, *Commentaries on the Laws of England, Book 4* (Lippencott & Co 1865) 190.

10 Blackstone, Book 2 (n 9) 429; Coke (n 9) 203; *Haynes’s Case* (1614), 12 Co Rep 113, 77 ER 1389 (Leicester Assizes) (E&W).

11 Heather Conway, *The Law and the Dead* (Routledge 2016); Imogen Jones and Muireann Quigley, ‘Preventing Lawful and Decent Burial: Resurrecting Dead Offences’ (2016) 36 Legal Studies 354; Stephen White, ‘The Laws Relating to Dealing with Dead Bodies’ (2000) 4 Medical Law International 145.

12 Conway (n 11); see eg, *Stewart* (n 5); also see cases on costs of burial, for eg, *Ambrose v Kerrison* (1851), 138 ER 307, 10 CB 776 (Common Pleas) (E&W); *Jenkins v Tucker* (1788), 126 ER 55, 1 H BL 90 (Common Pleas) (E&W); *Tugwell v Heyman* (1812) 70 ER 1389, 3 Camp 298 (Common Pleas) (E&W).

13 Michael Hirst, ‘Preventing the Lawful Burial of a Body’ (1996) Criminal Law Review 96; Imogen Jones, ‘A Grave Offence: Corpse Desecration and the Criminal Law’ (2017) 37 Legal Studies 599; Jones and Quigley (n 11); See eg, *R v Clark* (1883), 15 Cox C.C. 171 (Durham Assizes) (E&W); *R v Cundick* (1822), 171 ER 900, Dow & Ry NP 13 (Surrey Assizes) (E&W); *R v Fox and Ors* (1841), 2 WB 246, 114 ER 95 (Queen’s Bench) (E&W); *R v Newscomb* (1898), 2 CCC 255, 1898 CarswellNS 110 (County Court) (Nova Scotia); *Vann* (n 5).

14 See eg, *R v Gilles* (1820) Russ & Ry 366 at fn (b) (Northumberland Lent Assizes) (E&W); *R v Lynn* (1788), 2 TR 733, 100 ER 394, 395 (King’s Bench) (E&W); *R v Sharpe* (1857), Dears & Bell 162, 169 ER 959 (Court of Criminal Appeal) (E&W).

15 MacDonald (n 1); Richardson (n 1).

16
*R v Price* (1884), 12 QBD 247 (E&W).

17 See eg, in Canada, *Curry v Curry*, 2023 NSSC 402, para 44 (Supreme Court) (Nova Scotia); *Saleh v* Reichert, 1993 CanLII 9394, 104 DLR (4th) 384, paras 10, 14, 25 (Superior Court of Justice) (Ontario). Such comments sometimes stress the ‘no property rule’, as that was expressed again in *Williams v Williams* (1882) 20 Ch D 659 (High Court) (E&W), leaving the premise of decent burial intact unless legislation provides another way. *Contra*, see suggestions on the legality of tissue transplants and anatomy at common law in Jean-Gabriel Castel, ‘Some Legal Aspects of Human Organ Transplantation in Canada’ (1968) 46 Canadian Bar Review 345, 379–383.

18
*Phillips v Montreal General Hospital* (1908), 33 Que SC 483, 1908 CarswellQue 8 (Superior Court) (Québec); *Price* (n 16); Report of Board of Inquiry *Re Dr Ramsay Smith* (1903), GMG 24/6/0/498/1005-1903 (City of Adelaide Archives); also see James Brooke Little, *The Law of Burial* (Shaw and Sons 1894) 11; Arthur Turnour Murray, *The Law of Hospitals, Infirmaries, Dispensaries, and Other Kindred Institutions Whether Voluntary or Rate-Supported* (J. Murray 1908) 45; E Lewis Thomas (ed) *Baker’s Law Relating to Burials* (Sweet and Maxwell 1901) 36. Also see Cyril J Polson, *Disposal of the Dead* (Philosophical Library 1953) where Polson admits its lawfulness is the view of some.

19 See eg, *An Act to Regulate and Facilitate the Study of Anatomy 1843*, 7 Vic c 5 (United Canadas); *An Act Respecting the Study of Anatomy 1870*, 33 Vic c 3 (Nova Scotia).

20
*Re JS (Disposal of Body)*, [2016] EWHC 2859 (Fam), para 16 (High Court) (E&W).

21 Margaret Brazier, *Law and Healing: A History of a Stormy Marriage* (University of Manchester Press 2023); Margaret Brazier and Jonathan Montgomery, ‘Whence and Whither “Modern Medical Law”?’ (2019) 70 Northern Ireland Law Quarterly 5.

22 Brazier and Montgomery (n 21) 10.

23 Joshua Getzler, ‘Legal History as Doctrinal History’ in Markus D Dubber and Christopher Tomlins (eds), *The Oxford Handbook of Legal History* (Oxford University Press 2018) 171–192, 171.

24 ibid.

25 Brazier and Montgomery (n 21).

26 MacDonald (n 1); Richardson (n 1).

27 Richardson (n 1).

28 ibid; MJ Durey, ‘Bodysnatchers and Benthamites: The Implications of the Dead Body Bill for the London Schools of Anatomy, 1820-42’ (1976) 2 The London Journal 200.

29
*R v Davies and Another* (1828) (Lancaster Assizes) (E&W) Unreported; *R v Wilkinson and Young* (1785) (King’s Bench) (E&W) Unreported; also see House of Commons, *Report from the Select Committee on Anatomy* (1828) 6–7.

30
*Phillips* (n 18); *Price* (n 16); *Re Ramsay Smith* (n 18).

31 Brooke Little (n 18) 11; Lewis Thomas (n 18) 36; Turnour Murray (n 18) 45.

32 Contemporary comments have not described such a power, see eg, Conway (n 11). However, Stephen White (n 11) 154, hints at its possibility when referencing Jeremy Bentham’s dissection prior to the *Anatomy Act 1832*.

33
*Price* (n 16).

34 ibid 250.

35 ibid.

36 ibid 252–253; also see *Stewart* (n 5); *Vann* (n 5).

37
*Price* (n 16) 253.

38 ibid.

39 ibid; *Anatomy Act 1832* (n 4).

40
*Price* (n 16) 251.

41 ibid 252.

42 ibid.

43 ibid 254–255.

44 Brooke Little (n 18) 11; Lewis Thomas (n 18) 36; Turnour Murray (n 18) 45.

45 Lewis Thomas (n 18) 36.

46 ibid.

47 Brooke Little (n 18) 11.

48 Turnour Murray (n 18) 45.

49 ibid.

50 ibid.

51 ibid 46–50; *Anatomy Act 1832* (n 4); *Anatomy Act 1871*, 34 and 35 Vic c 16 (UK).

52
*Phillips* (n 18) para 13. These arguments are distinct from the suggestion that physicians may be entitled to dissect a body owing to the authority conferred by the coroner’s ancient powers at common law, see eg, *Davidson v Garrett* (1899), 30 OR 353, 5 CCC 200 (High Court) (Ontario).

53
*Phillips* (n 18) para 12.

54 ibid paras 8–12. The Court cites Sir J Fitz-James Stephen with apparent approval: ‘I suppose […] that anatomical specimens and the like are personal property’. See James Fitz-James Stephen, *History of Criminal Law of England* (William S. Hein 1883).

55
*Phillips* (n 18) para 17.

56 ibid para 16.

57 ibid para 17.

58 Cressida Fforde, *Collecting the Dead: Archaeology and the Reburial Issue* (Bristol Classic Press 2004); Marc Trabsky, *Law and the Dead: Technology, Relations and Institutions* (Routledge 2019).

59
*Re Ramsay Smith* (n 18).

60 ibid.

61 Fforde (n 58).

62
*Re Ramsay Smith* (n 18).

63 ibid.

64 ibid 1.

65 ibid; also see *Anatomy Act 1884*, 47 and 48 Vic 317 (South Australia).

66
*Re Ramsay Smith* (n 18) 1.

67 ibid; also see *Anatomy Act 1832* (n 4).

68
*Re Ramsay Smith* (n 18) 2.

69 ibid.

70 ibid.

71 ibid.

72 ibid 3–4.

73 Board of Inquiry, Notes of Evidence (1903), GMG 24/6/0/498/1005-1903 (Adelaide Archives), 39, 50–51, 53–54. Evidence came from witnesses John Lewis (or Louis) Dyer (Mortuary Attendant), William Lennox Cleland (Colonial Surgeon and Resident Medical Officer at the Parkside Lunatic Asylum), and Charles John Dolley (Mortuary Attendant).

74 ibid 151. Evidence came from witness Arthur Francis Lynch (Pathologist). Also see Richard Sanders Rogers (Member of Board of Management of Adelaide Hospital) 219. One witness, Dr Harry Swift (General Practitioner), testified contrarily at 220; however, upon examination by the Board and in cross examination the quality of his testimony was brought into doubt having regard to bias and contradictions at 226.

75 ibid 150. Evidence came from witness Arthur Francis Lynch (Pathologist). Also see Dr Edward Angas Johnson (Curator of the Pathological Museum, and Demonstrator of Anatomy) 163.

76
*Re Ramsay Smith* (n 18).

77 Shaun McVeigh, ‘Afterword: Office and the Conduct of the Minor Jurisprudent’ (2015) 5 UC Irvine Law Review 499, 500.

78
*Haynes’s Case* (n 10).

79 Editorial, ‘The Provincial Lunatic Asylum’ (1851) The Upper Canada Journal of Medical, Surgical and Physical Science 378; Editorial, ‘The British American Journal’s Lecture on Medical Ethics’ (1852) The Upper Canada Journal of Medical, Surgical and Physical Science 419.

80 Notes of Evidence (n 73).

81
*Exelby v Handyside* (1749), 2 East PC 652 (Common Pleas) (E&W).

82 ‘History’ *The Scots Magazine*, vol 11 (Edinburgh, 1 December 1749) 598.

83 ‘London, Dec. 2’ (1749) 565 The Ipswich Journal 1; regarding a Dr Handyside’s marriage to Miss Touchet, ‘near relation’ to Lord Castlehaven, see ‘Marriages’ (1749) 543 Ipswich Journal 1. Also see Paul Matthews, ‘Whose Body? People as Property’ (1983) 36 Current Legal Problems 193, 202. Matthews raises the possibility that Handyside settled out of concern for his reputation, but questions why Handyside would not have agreed to settle before coming to court. Given the poor esteem in which anatomists were held by the public in the eighteenth and nineteenth centuries, it seems possible to me that Handyside may have reconsidered the costs of winning the suit in the crowded Westminster Hall, especially when Chief Justice Willes expressed his opinion of the law in such a decidedly favourable manner.

84 ‘London, December 2’, *Derby Mercury* (Derby, 1 December 1749) 3; ‘Historical Chronicle’ *Gentleman’s Magazine*, vol 19 (London, 1749) 568; ‘The Monthly Chronologer’ *The London Magazine*, vol 18 (London, 1749) 574.

85
*Exelby* (n 81) 652.

86 There is reference to *Exelby* (n 81) in the Nisi Prius Record Book of the Second Prothonotory (1749), CP 36/9 (National Archives, Kew), but all it states is: ‘Middx Ss Exelby agt Handyside Issue ff 7-7: Smith’. The case is not mentioned in the plea rolls, likely because the matter was settled—see Ipswich Journal (n 83); Derby Mercury (n 84); Gentleman’s Magazine (n 84); London Magazine (n 84). Accounts in newspapers are brief and do not specify what Chief Justice Willes said. See Matthews (n 83).

87 Matthews (n 83).

88 The power to dissect could also complement the work-and-skill exception to the no-property rule, established in *Doodeward v Spence*, [1908] HCA 45, 6 CLR 406 (High Court) (Australia), as a precondition to the labour necessary to transform a mere corpse into a candidate for property.

89 Notes of Evidence (n 73).

90 Remigius Nwabueze, ‘Regulation of Bodily Parts: Understanding Bodily Parts as a Duplex’ (2019) 15 International Journal of Law in Context 515. Likewise, Nwabueze argues that the duplex character extends to the work-and-skill exception.

91
*Phillips* (n 18); *Edmonds v Armstrong Funeral Home Ltd*, 1930 CanLII 248, [1931] 1 DLR 676 (Court of Appeal) (Alberta); *R v Redmond* (1818) (Guildhall) (London) Unreported; regarding no need for consent with coronial examinations, see *Davidson* (n 52).

92
*Phillips* (n 18); *Edmonds* (n 91); *Redmond* (n 91). The case of *Redmond* is described in ‘Extraordinary Case’, *Morning Advertiser* (London, 7 December 1818) 4, ‘Dissecting Dead Bodies in Hospital’, *Exeter Flying Post*, vol 62 (Exeter, 17 December 1818) 2, and ‘Dissecting Dead Bodies in Hospital’, *Caledonian Mercury* (21 December 1818) 4. Unfortunately, I could not find legal records of *Redmond* in the London Archives. There is a paucity of Guildhall Justice Room records for this period, likely as a result of a fire in 1838.

93
*Edmonds* (n 91) 178.

94 ibid 179.

95 ibid 174.

96
*Morning Advertiser* (n 92) 4.

97 ibid.

98 ibid.

99 ibid.

100 ibid.

101 ibid.

102 ibid.

103 ibid.

104 Common law courts rarely acknowledge such interests in next of kin, possessory or otherwise, but see eg, *Miner v Canadian Pacific Railway Co.* (1910), 18 WLR 476, 3 Alta LR 408 (Supreme Court) (Alberta); *Bastien v Ottawa Hospital (General Campus)*, 2001 CanLII 28016, 56 O.R. (3d) 397 (Superior Court) (Ontario); *In Re Organ Retention Group Litigation*, [2004] EWHC 644, [2005] QB 506 (High Court) (E&W). Where the power is extant in the contemporary common law, arguably its exercise would be informed by prevailing norms around familial interest.

105
*Price* (n 16) 254–255.

106 ibid.

107 ibid 255.

108 ibid.

109 ibid.

110 ibid 256.

111
*Vade’s Case* (1818) (Mansion-House) (London) Unreported, see ‘Mansion-House’ *Johnson’s Sunday Monitor*, (London, 3 May 1818) 4. Unfortunately, I could not find legal records of the dispute in the London Archives. There is a paucity of Mansion-House Justice Room records for this period.

112
*Johnson’s Sunday Monitor* (n 111) 4.

113
*Public Health Act 1936*, 1936 c 49, s 161 (E&W); *Public Health (London) Act 1936*, 1936 c 50, s 296 (E&W).

114 Alfred Fellows, *The Law of Burial and Generally of the Disposal of the Dead* (Hadden, Best and Co. 1940) 84–86.

115
*Public Health Act 1875*, 1875 c 55, s 142 (E&W).

116 Fellows (n 114) 84–86.

117
*R v Parrott* (1855) [Unreported] (Surrey Quarter Sessions) (E&W), see ‘Indictments, 1855, Indictment 620a’, QS2/7/1855 (Surrey History Centre); ‘Minute Book, Court 2’, QS2/2/45 (Surrey History Centre).

118 ‘Unlawfully Anatomising a Dead Body’, *Lloyd’s Weekly Reporter* (London, 11 November 1855) 7; Editorial, ‘The Anatomy Act—Conviction for Illegality’ in Thomas Wakley (ed), *The Lancet, MDCCCLV*, Vol. 2 (1855) 443.

119
*The Lancet* (n 118).

120 Indictments (n 117); *Minute Book* (n 117). *The Lancet* (n 118) and *Lloyd’s Weekly Reporter* (n 118) appear to exaggerate the offence for which Dr Parrott was convicted, as the second count described in the indictment focused on the failure to notify the Anatomy Inspector. The minute book, which recorded the conviction, also only indicated the failure of notice.

121
*Re Stevenson and Others* (1877) (Glasgow Sheriff’s Court) (Scotland) Unreported, see ‘The Maternity Hospital Case’, *Glasgow Herald* (Glasgow, 13 October 1877) 3; ‘The Maternity Hospital Case’, *Glasgow Herald* (Glasgow, 23 October 1877) 7. Also see *Anatomy Act 1832* (n 4) s 15.

122
*Glasgow Herald* (13 October 1877) (n 121).

123 Peter Linebaugh, ‘The Tyburn Riots Against the Surgeons’ in Douglas Hay and others (eds), *Albion’s Fatal Tree: Crime and Society in Eighteenth-Century England* (Verso 1975) 65–117; also see Richardson (n 1).

124 Linebaugh (n 123); Richardson (n 1).

125 See eg, ‘Livery and Freemen of the Company of Barbers and Surgeons to the King Petition’, (1715 or 1716) SP 35/2/48 (National Archives, Kew); ‘Petition of the Company of Barbers and Surgeons’, (1719) SP 35/19/57 (National Archives, Kew).

126 Richardson (n 1). Physicians and surgeons were not, however, convicted unless they were involved in disinterring dead bodies. See eg, *Lynn* (n 14); *R v Cooke* (1827) (Devon Assizes) (E&W) Unreported, see Crown Roll, 7&8 Geo IV, Hilary Term (1827), Rolls 62 and 63, KB 28/501 (National Archives, Kew); *R v Davies and Another* (1828) (Lancaster Assizes) (E&W) Unreported, see House of Commons (n 29) 148; *R v Wilkinson and Young* (1785) (King’s Bench) (E&W) Unreported, see Crown Roll, 25 Geo III, Hilary Term (1785), Rolls 24 and 25, KB 28/332 (National Archives, Kew).

127 Richardson (n 1).

128 See eg, ‘Memorial of physicians and surgeons regarding importance of having bodies to dissect’, (1825) HO 44/15/78 (National Archives, Kew) ff 83–83a; House of Commons (n 29) 6–7.

129 House of Commons (n 29).

130 ibid 6–7; however, Thomas Halls, a Bow-street magistrate, thought dissection was not an offence at common law, 93–95; also see *Davies* (n 126); *Wilkinson and Young* (n 126).

131 The Court of King’s Bench in *Lynn* (n 14) 394–395, briefly raised the ‘case of one Young a few years ago’ to query whether it made any difference if the body was taken up for dissection or ‘indecent exhibitions’. Counsel arguing in favour of the motion to arrest the judgment, Sir William Garrow, distinguished the case by observing that there the ‘master of Shoreditch workhouse, a surgeon, and another person were indicted for conspiracy to prevent the burial of a person who died in the workhouse’. Garrow was also involved in *Wilkinson and Young* (n 126) for the prosecution, see *The New Annual Register or General Repository of History, Politics, and Literature for the Year 1785* (G.G.J. & J. Robinson 1786) 33. With respect to *Davies* (n 126), the case was heard a month before the Select Committee’s hearings in the UK. A document entitled ‘Extract from a Report of the Trial of John Davies and others’, printed by E Smith and Co. in Liverpool, formed an appendix to the Select Committee’s final report and was relied on as evidence, but reasons for the decision are not otherwise extant. See House of Commons (n 29) 148.

132 House of Commons (n 29) 6–7.

133 ibid 6.

134 Rolls 24 and 25 (n 126); Files of Indictments, etc (1783–1784), 24 Geo III, Michaelmas, Indictments 57 and 58, KB 10/43 (National Archives, Kew). Also see description in *New Annual Register* (n 131) 16 and 33; ‘London, Feb. 19’, *Stamford Mercury* (Stamford, 25 February 1785) 2; ‘Wednesday’s Post’, *Ipswich Journal* (Ipswich, 26 February 1785) 2; ‘Tuesday’s Post’, *Oxford Journal* (Oxford, 26 February 1785) 1.

135 Rolls 24 and 25 (n 126). The indictments emphasized that the deceased were ‘poor impotent person[s] unable to maintain and provide for [themselves]’ and, accordingly, under the superintendence and care of Wilkinson whose office required certain duties. Wilkinson was appointed to his office under the *Shoreditch (Poor Relief) Act 1774*, 14 Geo 3 c 29 (E&W), which established and maintained a workhouse and burial ground in the parish overseen by Trustees of the Poor. Wilkinson’s office appears to have been a defining part to at least some of the indictments, for it bestowed obligations that his and Young’s actions and conspiracies to act failed.

136 That seems to accord with Garrow’s distinction in *Lynn* (n 14) 395, and the King’s Bench appears to have accepted this distinction as there is no further mention of the case.

137
*Davies* (n 126); House of Commons (n 29) 148, 150.

138 House of Commons (n 29) 148.

139 ibid 150.

140 ibid 148. Evidence against others was either non-existent or ‘very slight’.

141 ibid 148, 150.

142 ibid 151.

143 ibid.

144 See eg, *Cundick* (n 13); *Clark* (n 13); *Sharpe* (n 14). See more in Jones and Quigley (n 11).

145 House of Commons (n 29) 8.

146 MacDonald (n 1); Richardson (n 1). Public feeling calmed but remained excitable as Richardson shows. The *Anatomy Act 1832* played a part in managing public feeling, by eliminating the raiding of churchyards (which were visible to family and friends) and replacing it with an institutional process that better concealed how bodies were procured.

147 Helen MacDonald, ‘Procuring Corpses: The English Anatomy Inspectorate, 1842 to 1858’ (2009) 53 Medical History 379. Also see Helen MacDonald, ‘A Scandalous Act: Regulating Anatomy in a Settler Colony, Tasmania 1869’ (2007) 20 Social History of Medicine 39.

148
*Price* (n 16) at 253; *Re Ramsay Smith* (n 18). Such claims seem to ignore that the Court of Assistants of the Company of Barbers and Surgeons of London disciplined a member—Mr William Cheseldon—in 1714 for holding private lectures over dissections and ‘[drawing] away members of this Company and others from [the Company’s] Public Dissections and Lectures at the [Surgeon’s] Hall’. Similar discipline was used previously against another member in 1573 for ‘havinge an Anathomye in his howse contrary to an order’. See James Blake Bailey, *The Diary of a Resurrectionist, 1811-1812* (Swan Soneschein & Co. 1896) 18–19. However, a distinction might be drawn between orders and bylaws applicable to members so not to interfere with the Company’s right to hold lectures, and privileges to conduct dissections generally.

149 Durey (n 28); Richardson (n 1).

150 ‘A Bill for Regulating Schools of Anatomy’, *The Lancet*, vol 17 (31 December 1831) 481, 484.

151 Cases with Counsel’s Opinion, 1746–1855, RCS-GOV/2/7/9 (Royal College of Surgeons of England) 152.

152
*Re Ramsay Smith* (n 18).

